# A phase I study to evaluate the effect of hepatic impairment on the pharmacokinetics and safety of suraxavir marboxil: a novel oral antiviral for influenza

**DOI:** 10.1128/aac.01668-25

**Published:** 2026-03-23

**Authors:** Jing Zhou, Shousheng Yan, Yan Zhao, Hong Zhang, Nanya Wang

**Affiliations:** 1 Phase I Clinical Research Center, The First Hospital of Jilin University 117971, Changchun, Jilin, China; 2 Jiangxi Qingfeng Pharmaceutical Co. 702539 , Ganzhou, Jiangxi, China; Chinese Academy of Medical Sciences & Peking Union Medical College, Beijing, China

**Keywords:** hepatic impairment, pharmacokinetics, safety, suraxavir, influenza

## Abstract

Suraxavir marboxil (GP681, abbreviated as suraxavir) is a novel, orally active small-molecule polymerase acidic (PA) inhibitor for the treatment of influenza. Its active metabolite, GP1707D07, specifically inhibits PA endonuclease activity. Suraxavir has been approved for use in adolescents (≥12 years) and adults with uncomplicated influenza A and B. This non-randomized, open-label, parallel-group phase I clinical trial evaluated the effects of hepatic impairment on the pharmacokinetics (PK) and safety of suraxavir. Subjects with mild or moderate hepatic impairment and matched healthy controls (*n* = 8 per group) received a single oral dose of 40 mg suraxavir. PK parameters, including maximum plasma concentration (C_max_), area under the plasma concentration–time curve from time 0 to last time point of measurable concentration (AUC_0–*t*_), and area under the plasma concentration–time curve from time 0 to infinity (AUC_0–∞_), were compared. Compared with subjects with normal hepatic function, the least-squares geometric mean ratios (90% CI) of GP1707D07 in subjects with mild hepatic impairment were 101.50% (72.63%–141.83%) for C_max_, 86.98% (69.80%–108.38%) for AUC_0–*t*_, and 87.06% (70.57%–107.40%) for AUC_0–∞_. In subjects with moderate impairment, the corresponding ratios were 203.63% (147.50%–281.13%) for C_max_, 155.23% (129.11%–186.64%) for AUC_0–*t*_, and 157.26% (131.39%–188.22%) for AUC_0–∞_. Mild impairment had minimal effect on GP1707D07 exposure, while moderate impairment increased C_max_ and AUC by ~1-fold and 0.5-fold, respectively, without resulting in clinically significant changes. A single 40-mg dose of suraxavir was safe and well tolerated in subjects with mild or moderate hepatic impairment. No dose adjustment is required for these populations.

**CLINICAL TRIALS:**

This study is registered with ClinicalTrials.gov as NCT05814926.

## INTRODUCTION

Influenza is an acute viral respiratory disease caused by influenza viruses (seasonal influenza A and B) infecting the human respiratory tract. Its clinical severity varies, and it can lead to outbreaks or pandemics, imposing a substantial global health burden (1–3). Globally, an estimated 1 billion influenza cases occur annually, including 3–5 million severe cases, with 290,000–650,000 deaths from influenza-associated respiratory disease (4). Populations at high risk, including young children, the elderly, pregnant women, immunocompromised individuals, and patients with severe underlying conditions, are more likely to progress to severe or critical illness (5–7).

Currently, two major classes of anti-influenza drugs are available: M2 ion channel inhibitors (e.g., amantadine and rimantadine) and neuraminidase inhibitors (NAIs, e.g., oseltamivir and zanamivir). However, influenza viruses—particularly influenza A virus—can rapidly develop resistance through mutations, significantly undermining treatment efficacy. Due to issues of resistance and safety, M2 inhibitors are no longer recommended, and resistance to NAIs has also emerged (8–12). Thus, new antiviral agents with improved efficacy and novel mechanisms of action are urgently needed.

Influenza virus RNA polymerase has emerged as a promising therapeutic target. Baloxavir marboxil, a polymerase acidic (PA) endonuclease inhibitor, demonstrated significant efficacy compared with placebo in shortening symptom duration and reducing secondary transmission among household contacts (13–15). Baloxavir marboxil also showed superiority to oseltamivir in alleviating influenza symptoms, lowering viral load, and reducing adverse events (AEs), particularly in pediatric populations, with the advantage of single-dose administration (16–18). However, the emergence of resistant strains has limited its clinical benefit (19, 20), underscoring the need for safer and more effective antivirals with a high genetic barrier to resistance.

Suraxavir marboxil (GP681, abbreviated as suraxavir) is a novel, orally active small-molecule PA inhibitor whose active metabolite, GP1707D07, specifically inhibits PA endonuclease activity, thereby blocking influenza viral replication. *In vitro*, suraxavir exhibited nanomolar antiviral activity against both influenza A and B viruses and demonstrated approximately 1,000-fold greater potency than oseltamivir. The mean half-maximal inhibitory concentration (IC_50_) for inhibition of viral transcription was 0.6 nM. By comparison, the mean IC_50_ of baloxavir was 1.8 nM. *In vivo*, suraxavir showed antiviral efficacy comparable to that of oseltamivir and baloxavir (21, 22). In a phase I trial (NCT04729764), single oral doses of 20–80 mg suraxavir were safe and well tolerated. Plasma concentrations in the 20-mg and higher dose groups exceeded the target therapeutic concentration (6.87 ng/mL) at 24 hours and were maintained for 72–136 hours (23). In a phase II trial (NCT04736758), single doses of 20–40 mg suraxavir significantly reduced time to symptom resolution compared with placebo in adults with uncomplicated influenza, with good safety, establishing 40 mg as the optimal dose (21). In a phase III trial (NCT05474755), a single 40 mg dose significantly shortened symptom duration compared with placebo and was well tolerated (22). Suraxavir has now been approved for clinical use as a broad-spectrum, effective, single-dose oral therapy for influenza, expected to improve both treatment efficacy and patient adherence.

Post-marketing, suraxavir will also be used in patients with mild-to-moderate hepatic impairment. Preclinical data indicate that cytochrome P450 (CYP) enzymes, including CYP1A2, CYP2B6, CYP2C8, and CYP3A4, are involved in the metabolism of GP1707D07, and the liver is a potential target organ for toxicity (22). Hepatic impairment may alter drug metabolism, plasma protein binding, and biliary excretion, potentially reducing clearance and impacting both safety and efficacy (24, 25). Patients with severe underlying liver disease infected with influenza virus are at increased risk of developing severe or critical illness. In accordance with the U.S. Food and Drug Administration (FDA) guidance on pharmacokinetic studies in patients with hepatic impairment (26, 27), it is necessary to investigate the pharmacokinetics and safety of suraxavir in this population to determine whether dose adjustment is required. The present study aimed to evaluate the pharmacokinetics, safety, and tolerability of a single oral dose of 40 mg of suraxavir in subjects with mild or moderate hepatic impairment compared to a matched healthy control group.

## MATERIALS AND METHODS

### Study design

This multicenter, open-label, single-dose, parallel-group clinical trial was conducted to evaluate the pharmacokinetics (PK) and safety of a single 40-mg oral dose of suraxavir in subjects with mild or moderate hepatic impairment compared with healthy controls (ClinicalTrials.gov identifier: NCT05814926). A total of 24 subjects were enrolled and assigned to 3 groups (*n* = 8 each): mild hepatic impairment (Child-Pugh class A), moderate hepatic impairment (Child-Pugh class B), and healthy controls. Healthy subjects were matched to impaired subjects by sex (±1 subject), mean age (±15 years), and mean body weight (±25%).

Screening assessments were performed within 28 days prior to dosing (day −28 to day −1). Eligible participants were admitted to the study center 1 day before dosing, received a standardized light evening meal, and fasted for at least 10 hours overnight. On day 1, subjects received a single 40-mg oral dose of suraxavir with 240 mL of water under fasting conditions. Drinking water was prohibited from 1 hour before to 1 hour after dosing. Standardized meals were provided 4 hours (lunch) and 10 hours (dinner) post-dose. Physical activity was restricted within 2 hours after dosing. Subjects remained in the study center for scheduled PK sampling and safety assessments through day 6. Thereafter, they were either discharged on day 6 with outpatient follow-up visits on days 8 and 12 or remained hospitalized until day 12 as per investigator discretion.

Safety assessments included monitoring of adverse events, vital signs, physical examinations, clinical laboratory tests (hematology, serum biochemistry, urinalysis, and coagulation), 12-lead electrocardiograms, and concomitant medication use.

### Study participants

Eligible participants met the following inclusion criteria: adults aged 18–68 years, male or female; body weight ≥50 kg for males and ≥45 kg for females; body mass index (BMI) between 18 and 30 kg/m². Participants with hepatic impairment were required to have a documented history of primary liver disease and a Child-Pugh classification of grade A (scores 5 and 6) or grade B (scores 7–9). Patients either had not received any medications within 4 weeks prior to screening or, if requiring long-term therapy for hepatic dysfunction and/or other comorbidities, were on a stable regimen for at least 4 weeks. Healthy controls with normal hepatic function were matched to participants with hepatic impairment by age, sex, and body weight. At screening, laboratory safety tests had to be within normal limits or show only mild abnormalities judged not clinically significant by the investigator.

The primary exclusion criteria were use of herbal medicines, strong cytochrome P450 (CYP450) enzyme inhibitors, or strong inducers within 4 weeks prior to dosing; clinically significant abnormal blood pressure; clinically significant electrocardiographic abnormalities, including tachycardia or bradycardia requiring treatment, second- or third-degree atrioventricular block, or corrected QT interval using Fridericia’s formula (QTcF) prolongation (QTcF >450 ms in males and >460 ms in females); participation in another clinical trial with investigational drug exposure within 3 months; blood loss or donation ≥400 mL within 3 months before dosing; pregnancy, lactation, or positive pregnancy test. Additional exclusion criteria applied to participants with hepatic impairment: history of liver transplantation, drug-induced liver injury, acute hepatic dysfunction of any etiology, hepatic failure, or cirrhosis complicated by hepatic encephalopathy, hepatocellular carcinoma, esophagogastric variceal bleeding, or other conditions deemed unsuitable by the investigator. For participants with normal hepatic function, exclusion criteria included a history of significant primary organ disease or any prior history of hepatic dysfunction.

### Pharmacokinetic assessments

Blood samples for pharmacokinetic (PK) evaluation were collected at 17 time points: within 1 hour prior to dosing on day 1 and at 0.5, 1.0, 2.0, 3.0, 4.0, 5.0, 6.0, 8.0, 12.0, 24.0, 36.0, 48.0, 72.0, 120.0, 168.0, and 264.0 hours post-dose. At each time point, 4 mL of peripheral venous blood was drawn into tubes containing sodium fluoride/potassium oxalate as anticoagulants. Samples were gently inverted five to eight times and placed upright in an ice-water bath. Within 1 hour after collection, blood samples were centrifuged at 1,500 g for 10 minutes at 2°C–8°C. Plasma was separated and aliquoted within 1 hour after centrifugation, then stored at ≤−60°C until analysis. Plasma concentrations of suraxavir and its metabolite GP1707D07 were quantified using a validated high-performance liquid chromatography tandem mass spectrometry method. The concentration range for the determination of suraxavir and its active metabolite GP1707D07 in human plasma was 0.3–300 ng/mL. The bioanalytical method validation report was archived at Frontage Laboratories (Suzhou, China) Co., Ltd. Intra- and inter-batch precision and accuracy of quality control samples met regulatory acceptance criteria, and reproducibility of the bioanalytical assays was confirmed.

### Safety assessments

Safety evaluations included monitoring of adverse events (AEs), vital signs, physical examinations, clinical laboratory tests (hematology, serum biochemistry, urinalysis, and coagulation tests), and 12-lead electrocardiograms. For each AE, severity, onset time, resolution time, duration, management, and outcome were recorded, and the relationship to the study drug was determined. The severity of AEs was graded according to the National Cancer Institute Common Terminology Criteria for Adverse Events, version 5.0.

### Statistical analysis

Non-compartmental analysis of plasma concentration-time data was performed using Phoenix WinNonlin version 8.3 to estimate PK parameters. Primary PK parameters included maximum plasma concentration (C_max_), area under the concentration–time curve from time 0 to the last time point of measurable concentration (AUC_0*–t*_), and area under the plasma concentration–time curve from time 0 to infinity (AUC_0–∞_). Secondary PK parameters included, but were not limited to, time to reach C_max_ (T_max_), elimination half-life (*t*_1/2_), terminal elimination rate constant (λ_*z*_), apparent volume of distribution after oral dosing (*V*_*z*_/*F*), apparent clearance after oral dosing (CL/*F*), and mean residence time (MRT). C_max_, AUC_0–*t*_, and AUC_0–∞_ values were log transformed and analyzed using analysis of variance, with hepatic function group as a fixed effect. Geometric mean ratios (hepatic impairment/normal hepatic function) and the corresponding 90% CIs were calculated. If the 90% CIs of the geometric mean ratios for C_max_, AUC_0–*t*_, and AUC_0–∞_ in participants with mild or moderate hepatic impairment fell within the predefined bioequivalence range of 80.00%–125.00%, no significant difference was concluded between the impaired and normal hepatic function groups. Differences in T_max_ among hepatic function groups were evaluated using the Wilcoxon rank-sum test. Statistical analyses were conducted using SAS version 9.4. In addition, exploratory analyses were performed to assess potential correlations between PK parameters of suraxavir and GP1707D07 (e.g., AUC) and indices of hepatic function. Log-transformed AUC_0–*t*_, and AUC_0–∞_ values were considered dependent variables, while baseline albumin, total bilirubin, prothrombin time, and Child-Pugh classification were included as independent variables in linear regression models.

## RESULTS

### Participants

A total of 53 subjects were screened, and 24 subjects were enrolled and dosed, with 8 subjects in each group: mild hepatic impairment, moderate hepatic impairment, and normal hepatic function. Twenty-three subjects completed the study; one subject in the normal hepatic function group withdrew voluntarily on day 6 post-dose. Demographics and baseline characteristics, including age, sex, and body weight, were comparable across groups (Table 1).

**TABLE 1:** Baseline demographics and clinical characteristics^*a*^

Baseline parameter	Mild hepatic impairment (*N* = 8)	Moderate hepatic impairment (*N* = 8)	Normal hepatic function (*N* = 8)	Total (*N* = 24)
Age, years	53.1 ± 5.30	54.4 ± 6.05	50.8 ± 5.60	52.8 ± (5.62)
Sex, male/female	6/2	7/1	7/1	20/4
Weight, kg	68.44 ± 7.12	76.28 ± 8.38	65.34 ± 7.62	70.02 ± 8.751
BMI, kg/m^2^	25.83 ± 1.84	26.91 ± 2.52	24.05 ± 2.30	25.60 ± 2.46
Ethnicity, *n* (%)				
Han	6 (75.0)	7 (87.5)	8 (100)	21 (87.5)
Others	2 (25.0)	1 (12.5)	0	3 (12.5)
Child-Pugh score	5.4 (0.52)	7.1 (0.35)	NA^*b*^	NA

^*a*^ Values are means ± standard deviations, or numbers of subjects (%)；BMI, body mass index.

^*b*^ NA, not applicable.

### Pharmacokinetics

All eight subjects in each group were included in the PK parameter data set and C_max_ equivalence analysis. Due to the withdrawal of one subject in the normal hepatic function group, eight subjects each in the mild and moderate hepatic impairment groups and seven subjects in the normal group were included in the AUC_0–*t*_ and AUC_0–∞_ equivalence analysis.

**Fig 1:**
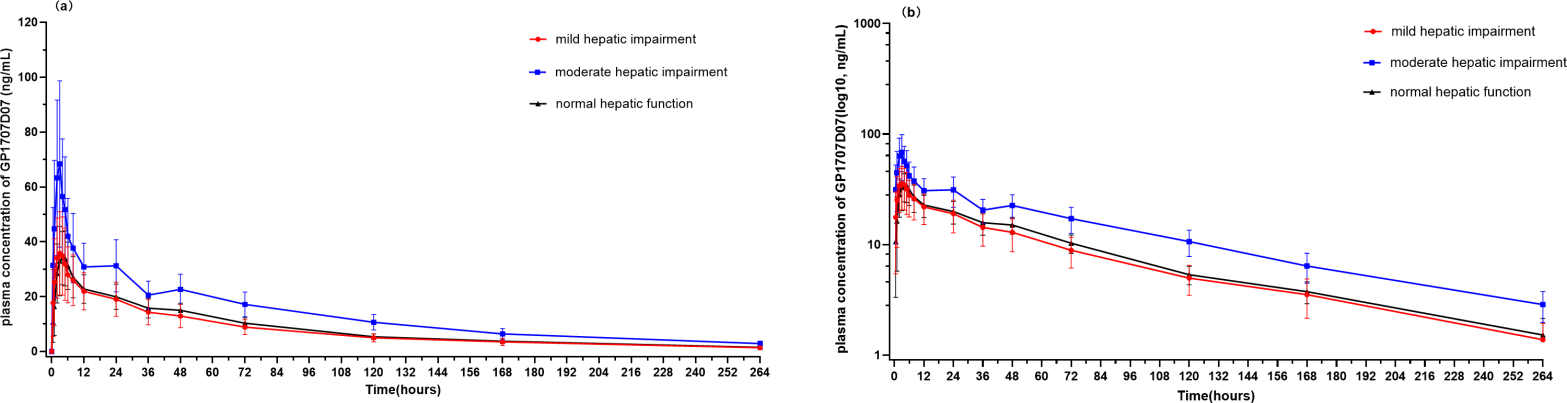
Mean plasma concentration-time profiles of GP1707D07, the active metabolite of suraxavir marboxil, in Chinese subjects with normal or impaired hepatic function following a single oral dose of 40 mg suraxavir marboxil: (**a**) linear scale and (**b**) semi-logarithmic scale.

Because suraxavir is rapidly metabolized *in vivo* to its active metabolite GP1707D07, plasma concentrations of suraxavir were largely below the lower limit of quantification, preventing calculation of additional PK parameters; therefore, PK analyses focused on GP1707D07 only. Mean plasma concentration–time curves and semi-logarithmic plots for GP1707D07 are shown in Fig. 1. The primary PK parameters are summarized in Table 2, and comparisons between hepatic impairment and normal groups are shown in Table 3.

**TABLE 2:** Pharmacokinetic parameters of GP1707D07^*a*^^,^^*b*^

PK parameters	Mild hepatic impairment (*N* = 8)	Moderate hepatic impairment (*N* = 8)	Normal hepatic function (*N* = 8)
C_max_ (ng/mL)	37.49 (38.06%)	75.09 (42.06%)	35.81 (29.79%)
AUC_0–*t*_ (h*ng/mL)	1967.51 (29.01%)	3468.57 (25.45%)	2071.53 (22.67%)
AUC_0–∞_(h*ng/mL)	2125.56 (27.22%)	3801.47 (24.23%)	2269.52 (19.56%)
T_max_ (h)	2.99 (2.00, 4.00)	3.00 (2.00, 3.00)	4.00 (3.00, 5.00)
*t*_1/2_ (h)	75.80 (29.25%)	77.54 (19.91%)	72.36 (30.96%)
λ_*z*_ (h^−1^)	0.010 (24.30%)	0.009 (19.43%)	0.010 (27.51%)
CL/*F* (L/h)	20.15 (28.23%)	11.02 (21.78%)	18.35 (23.32%)
*V*_*z*_/*F* (L)	2239.21 (45.04%)	1230.69 (27.25%)	1856.79 (27.30%)
MRT_0–*t*_ (h)	70.64 (14.79%)	75.84 (10.04%)	67.36 (18.63%)
MRT_0–∞_ (h)	94.87 (25.87%)	102.83 (19.43%)	92.12 (28.58%)

^*a*^ Data are expressed as geometric means (CV%), or median (minimum, maximum). MRT, mean residence time.

^*b*^ One subject in the normal hepatic function group withdrew on day 6 post-dose; available pharmacokinetic data from this subject were included in the pharmacokinetic parameter analysis.

**TABLE 3:** Ratios for pharmacokinetic parameters of GP1707D07 in subjects with hepatic impairment versus normal hepatic function^*a*^

Pharmacokinetic parameters	Ratio (%) (hepatic impairment/normal)	90% CI (%)
Mild hepatic impairment		
C_max_ (ng/mL)	101.50	72.63, 141.83
AUC_0−*t*_ (h*ng/mL)	86.98	69.80, 108.38
AUC_0−∞_ (h*ng/mL)	87.06	70.57, 107.40
Moderate hepatic impairment		
C_max_ (ng/mL)	203.63	147.5, 281.13
AUC_0−*t*_ (h*ng/mL)	155.23	129.11, 186.64
AUC_0−∞_ (h*ng/mL)	157.26	131.39, 188.22

^*a*^ One subject in the normal hepatic function group withdrew on day 6 post-dose; therefore, AUC_0−*t*_ and AUC_0−∞_ for this subject were not included in the equivalence analysis.

Plasma concentration-time profiles of GP1707D07 were generally consistent across groups. In participants with mild or moderate hepatic impairment, mean plasma concentrations peaked approximately 3 hours post-dose, whereas peak concentration in healthy subjects was delayed by 1 hour. Drug elimination was slow and comparable among groups, with mean *t*_1/2_ ranging from 72.36 to 77.54 hours. Compared with subjects with normal hepatic function, the least-squares geometric mean ratios (90% CI) of the active metabolite GP1707D07 in subjects with mild hepatic impairment were 101.50% (72.63%−141.83%) for C_max_, 86.98% (69.80%−108.38%) for AUC_0−*t*_, and 87.06% (70.57%−107.40%) for AUC_0−∞_. For subjects with moderate hepatic impairment, the corresponding ratios were 203.63% (147.50%−281.13%) for C_max_, 155.23% (129.11%−186.64%) for AUC_0−*t*_, and 157.26% (131.39%−188.22%) for AUC_0−∞_. Compared with healthy subjects, participants with mild hepatic impairment had similar C_max_ values, with AUC_0−*t*_ and AUC_0−∞_ reduced by approximately 13%. In participants with moderate hepatic impairment, C_max_ and AUC values were approximately 2-fold and 1.5-fold higher than those in healthy subjects, respectively (Table 3).

In the sensitivity analysis, all eight subjects in the normal hepatic function group were included in the bioequivalence set for the analysis of AUC_0−*t*_ and AUC_0−∞._ The geometric mean ratios (hepatic impairment/normal hepatic function) and corresponding 90% confidence intervals for AUC_0−*t*_ and AUC_0−∞_ were 93.95% (73.55%−120.02%) and 92.28% (74.07%−114.96%), respectively, for subjects with mild hepatic impairment. For subjects with moderate hepatic impairment, the corresponding geometric mean ratios and 90% confidence intervals were 167.68% (134.82%−208.54%) for AUC_0−*t*_ and 166.69% (137.10%−202.67%) for AUC_0−∞_. In the moderate hepatic impairment group, the geometric mean AUC values were approximately 1.6-fold higher than those observed in subjects with normal hepatic function. The results of the sensitivity analysis were generally consistent with those of the primary analysis.

Univariate analyses with linear regression models assessed correlations between individual hepatic function indices or Child-Pugh classification and PK parameters (AUC_0−*t*_, AUC_0−∞_). Results indicated that for each unit decrease in baseline albumin, AUC_0−*t*_ and AUC_0−∞_ increased by 0.0390 h·ng/mL (*P*=0.012 and 0.011, respectively). Each unit increase in baseline total bilirubin was associated with an increase of 0.0122 h·ng/mL (AUC_0−*t*_, *P*=0.023) and 0.0126 h·ng/mL (AUC_0−∞_, *P*=0.018). Each unit increase in baseline prothrombin time corresponded to an increase of 0.0816 h·ng/mL (AUC_0−*t*_, *P*=0.045) and 0.0881 h·ng/mL (AUC_0−∞_, *P*=0.029). Compared with mild hepatic impairment, participants with moderate hepatic impairment showed an average increase in AUC_0−*t*_ of 0.5793 h·ng/mL and in AUC_0−∞_ of 0.5913 h·ng/mL (both *P*<0.001).

### Safety and tolerability

Single-dose administration of 40 mg suraxavir was well tolerated in participants with mild or moderate hepatic impairment. No serious adverse events, study withdrawals due to AEs, or deaths occurred. Most AEs were grade 1 or 2 in severity. Hematologic abnormalities were the most common adverse events, with decreased white blood cell and neutrophil counts observed in 29.2% and 25.0% of subjects, respectively. Several grade 3 adverse events (AEs) occurred in participants with moderate hepatic impairment and were hematologic in nature. One subject experienced a grade 3 decrease in platelet count; two subjects experienced grade 3 decreases in lymphocyte count; and two subjects experienced grade 3 decreases in white blood cell and neutrophil counts. All grade 3 events resolved without medical intervention, and outcomes were recovery, improvement, or stable, except for a few participants who declined follow-up. A summary of all AEs is provided in Table 4. Incidence of AEs was 75% in both hepatic impairment groups and 37.5% in the normal hepatic function group. Most AEs were laboratory abnormalities of mild-to-moderate severity and resolved without treatment. Incidence of AEs was comparable between mild and moderate hepatic impairment groups and higher than in healthy controls.

**TABLE 4:** Treatment-emergent adverse events after administration of suraxavir marboxil^*a*^

Adverse events	Mild hepatic impairment (*N* = 8), *n (%*)	Moderate hepatic impairment (*N* = 8)*, n (%*)	Normal hepatic function (*N = 8), n (%*)
White blood cell count decreased	1 (12.5)	6 (75.0)	0
Neutrophil count decreased	1 (12.5)	5 (62.5)	0
Blood triglycerides increased	0	1 (12.5)	3 (37.5)
Platelet count decreased	2 (25.0)	2 (25.0)	0
Lymphocyte count decreased	0	3 (37.5)	0
Blood albumin decreased	0	2 (25.0)	0
Blood glucose increased	1 (12.5)	1 (12.5)	0
Alanine aminotransferase increased	0	1 (12.5)	0
High-density lipoprotein decreased	1 (12.5)	0	0
Urinary leukocyte positive	1 (12.5)	0	0
Protein urine present	0	1 (12.5)	0
Prothrombin time prolonged	1 (12.5)	0	0
Aspartate aminotransferase increased	0	1 (12.5)	0
Blood calcium decreased	1 (12.5)	0	0
Blood potassium decreased	1 (12.5)	0	0
Blood fibrinogen decreased	1 (12.5)	0	0
Blood pressure increased	1 (12.5)	0	0
Nausea	1 (12.5)	0	0
Diarrhea	1 (12.5)	0	0
Upper abdominal pain	0	1 (12.5)	0
Toothache	1 (12.5)	0	0
COVID-19 infection	1 (12.5)	1 (12.5)	0
Hyperuricemia	1 (12.5)	0	0
Rash	0	1 (12.5)	0
Pruritus	0	1 (12.5)	0
Anemia	0	1 (12.5)	0

^*a*^ The data are presented as *n* (%).

## DISCUSSION

Suraxavir is a novel PA inhibitor with improved efficacy and enhanced adherence due to its single-dose regimen, and it has been approved for the treatment of uncomplicated influenza A and B in adolescents and adults aged ≥12 years. Preclinical studies as well as phase I–III clinical trials have demonstrated that suraxavir effectively inhibits influenza virus replication, with substantial efficacy advantages and a favorable safety profile (21–23).

Because hepatic impairment may alter drug metabolism, reduce drug clearance, and consequently affect drug safety and efficacy (24, 25), and given that hepatic elimination is the primary route of suraxavir clearance, the present study evaluated the impact of mild and moderate hepatic impairment on plasma exposure to suraxavir in Chinese subjects, as well as its safety and tolerability in patients with mild or moderate hepatic impairment. The results of this study provide a basis for determining whether dose adjustment of suraxavir is required in patients with impaired hepatic function. Based on the dosing regimen used in the phase III study, a single dose of 40 mg was selected for this study. In accordance with the FDA Guidance for Industry: Pharmacokinetics in Patients With Impaired Hepatic Function (27), a minimum of eight subjects per group is considered sufficient to provide evaluable data. Therefore, this study enrolled eight subjects in each group, which meets the recommendations outlined in the guidance.

This study assessed the impact of mild and moderate hepatic impairment on suraxavir exposure in Chinese subjects, as well as the safety and tolerability of suraxavir in these populations. After single oral administration of 40 mg suraxavir, the drug was rapidly metabolized to the active metabolite GP1707D07 in all groups, with slow and comparable elimination (mean *t*_1/2_: 72.36–77.54 hours). Peak plasma concentration of GP1707D07 occurred approximately 1 hour earlier in participants with hepatic impairment compared with healthy subjects. Geometric mean ratios (90% CI) of C_max_, AUC_0–*t*_, and AUC_0–∞_ for mild and moderate hepatic impairment versus normal hepatic function were not fully contained within the conventional 80%–125% bioequivalence range. Mild hepatic impairment had minimal impact on AUC, whereas moderate hepatic impairment increased C_max_ and AUC by approximately 1-fold and 0.5-fold, respectively. According to FDA guidance, demonstrating maintenance of PK parameters within the 80%–125% range is challenging in small-scale hepatic impairment studies; when AUC increases more than or equal to twofold, hepatic impairment clearly affects PK, and dose adjustment is recommended. Therefore, no dose adjustment is required for mild or moderate hepatic impairment, consistent with findings for the PA inhibitor baloxavir marboxil, where PK is not clinically significantly altered in mild or moderate hepatic impairment (28).

Hepatic impairment can reduce albumin synthesis and CYP activity, affecting drug absorption and metabolism. This may decrease plasma protein binding, increase free drug concentration, and expand the apparent volume of distribution, resulting in earlier peak plasma concentration. These mechanisms are consistent with the observed PK parameters and exploratory analyses in this study.

In this study, systemic exposure to suraxavir was higher in subjects with moderate hepatic impairment than in those with mild impairment. This difference is most likely attributable to the greater degree of hepatic dysfunction in the moderate group, as reflected by higher Child-Pugh scores (7.1 versus 5.4), which is commonly associated with reduced hepatic metabolic capacity and hepatic blood flow, leading to decreased clearance and increased systemic exposure. Differences in the underlying etiology of liver disease may have further contributed to the observed variability, as subjects with mild hepatic impairment predominantly had chronic viral hepatitis, and none had alcoholic cirrhosis, whereas the moderate hepatic impairment group included three subjects with alcoholic cirrhosis. Alcohol-related liver disease is often associated with more pronounced structural liver damage and reduced activity of drug-metabolizing enzymes, which may further impair drug clearance beyond that indicated by Child-Pugh classification alone. However, given the limited sample size, larger studies are warranted to further characterize the impact of hepatic disease etiology on suraxavir pharmacokinetics.

The lower systemic exposure observed in the healthy control subjects in the present study, as reflected by reduced C_max_ and AUC values compared with those reported in a previous phase I study (23), is likely attributable to differences in demographic characteristics and study design. In the present study, healthy control subjects were age and weight matched to participants with hepatic impairment and were, therefore, older and heavier than the young healthy volunteers typically enrolled in phase I studies. Age-related changes in gastrointestinal absorption, hepatic blood flow, and higher body weight may have contributed to the lower plasma exposure observed. Such differences are expected in matched-control studies and do not affect the interpretation of the impact of hepatic impairment on the pharmacokinetics of suraxavir.

Safety and tolerability were favorable across all groups. Although participants with hepatic impairment experienced more AEs, no new safety signals were observed compared with prior phase I–III studies. All AEs were mild to moderate, with no serious AEs or discontinuations. Only one grade 1 elevation each in aspartate aminotransferase and alanine aminotransferase occurred, indicating no clinically significant hepatotoxicity. Hematologic AEs, such as decreased white blood cell, neutrophil, and lymphocyte counts, were more frequent in moderate hepatic impairment (75%, 62.5%, and 37.5%, respectively). This may reflect higher systemic exposure, as the drug could have an enhanced effect on the hematopoietic system at elevated concentrations. In addition, hepatic impairment itself is associated with a higher baseline risk of hematologic abnormalities, which may contribute to their more frequent occurrence in patients with moderate hepatic impairment and are not necessarily directly or solely caused by the drug. All events were mild or moderate, not associated with clinical complications, and did not lead to study discontinuation. These findings suggest that single-dose suraxavir does not cause clinically meaningful hematologic or hepatic toxicity in mild or moderate hepatic impairment, though monitoring may be warranted in patients with moderate or greater impairment.

The study’s limitations include a small sample size, which may limit generalizability, and exclusion of subjects with severe hepatic impairment (Child-Pugh class C) due to safety concerns. Exposure is expected to increase substantially in severe hepatic impairment, and similar PA inhibitors, such as baloxavir marboxil, have not been studied in this population.

### Conclusion

Single oral administration of 40 mg suraxavir demonstrated no clinically significant differences in pharmacokinetics between patients with mild or moderate hepatic impairment and subjects with normal hepatic function. The drug was well tolerated and exhibited a favorable safety profile. Therefore, no dose adjustment of suraxavir is required for patients with mild or moderate hepatic impairment.

## Data Availability

The data sets generated during and/or analyzed during the current study are available from the corresponding author on reasonable request.
